# Contagious Diseases and County Newspapers: Unsanitary Conditions of Towns

**Published:** 1903-11

**Authors:** W. H. Blythe

**Affiliations:** Mt. Pleasant, Texas


### Correspondence.

### Contagious Diseases and County Newspapers: Unsanitary Conditions of Towns.

MT. PLEASANT, TEXAS, October 10, 1903.

*Editor Texas Medical Journal, Austin, Texas.*

DEAR SIR: I wish to say a few words about county newspapers as regards their relation to the people of the county in which the paper is published.

If I mistake not, every newspaper has the privilege of mailing the paper to actual subscribers within the county free of any postage or expense of carriage in any way. The United States government has granted this favor to these newspapers so that all the citizens of the county may become better informed and acquainted on local and general news, especially local news.

During the past three years a great many county newspapers have omitted giving all county news, which news would have been of vital interest and benefit to the people in the rural districts, also to the trades people of the town. I refer to contagious diseases; the existence of which all people should know. But, in too many instances, the people have been kept in the dark from the fact that the papers have been silent upon the subject; particularly smallpox—and why? Simply because they have been interviewed by some number of men who pay occupation tax that permits them to merchandise in goods, wares, etc., etc.; but are persuaded to believe the tax permits them to trade in human life, whereby the goose that lays the golden egg may lose her life. In other words, threaten to boycott the publisher if he publishes any smallpox news. I have been told by the editors of two papers that they were threatened, by merchants, also two publishers of newspapers in one of our neighboring States made oath in court that they were threatened with financial loss, and further informed that any smallpox news in their paper would not be tolerated in any way by the merchants of their respective towns.

Now, if the United States government can grant this franking privilege within the county to all newspapers published within their respective counties for the purpose of informing the citizens upon subjects and information that is their right, there is a moral obligation as well as a legal one. In the neglect of thus performing the obligation on the part of the newspapers to the citizens, could not the United States government remedy it, at least so far as letting the paper pay postage?

There are thousands of pounds of newspapers each week sent out, through the postoffice, into the counties of Texas free of postage or expense to the publishers.

So, if these papers accept this favor from the government, could not the government exact of these newspapers, in fact all accepting and using this franking privilege, to publish in their regular weekly issues a full report for the county of all contagious diseases; the report to be obtained from the county judge, as reported to him by the county health officer, and in default the franking privilege be withdrawn from such papers?

Well, we are done with the newspapers. Now we wish to turn our attention to town and city governments. There is scarcely a town or city council that does not pay particular attention to having the streets kept clean, but lose all sight of the back yards of dwelling houses and of store houses, also drains containing filthy mud and water. When we stop to consider the wide spread of typhoid fever, we find it alarmingly great, affording, next to consumption, a fearful mortality. A large percentage of this disease could be prevented by our municipal authorities. If not by them—by reason of their lack of knowing how—then should they appoint a municipal health board who do know how, and whose duty it should be to have all unsanitary parts of the town put into a clean and sanitary condition and kept so.

Think of how many wells and cisterns are made the focus of cause of typhoid fever, when by a little exercise of law it could be prevented.

The strength of our nation lies in the health of our citizens. So let us try to clean and clear up our back yards and obtain free drainage.

Smallpox and typhoid fever are not all the diseases to be looked after, for we have others of equal importance. It appears, judging from the past, that our commissioners are not in full realization of the importance of sanitary work; so it becomes our duty to wage a warfare on all sources of danger—beginning at the county commissioners and on up to the Legislature. One citizen has no right to endanger the life or health of another citizen by an unsanitary condition of his premises, and when such conditions do exist, and they do, the law should be enforced against such offender. Clean up, clean up, or get out. Let all offenders of sanitary law be educated to a sense of duty to themselves and the balance of mankind.

W. H. Blythe, M. D.

